# (*E*)-1-(4-Methyl­phen­yl)ethanone [8-(trifluoro­meth­yl)quinolin-4-yl]hydrazone

**DOI:** 10.1107/S1600536810009475

**Published:** 2010-03-20

**Authors:** Grzegorz Dutkiewicz, Anil N. Mayekar, H. S. Yathirajan, B. Narayana, Maciej Kubicki

**Affiliations:** a Department of Chemistry, Adam Mickiewicz University, Grunwaldzka 6, 60-780 Poznań, Poland; b Department of Studies in Chemistry, University of Mysore, Mysore 570 006, India; c Department of Studies in Chemistry, Mangalore University, Mangalagangotri 574 199, India

## Abstract

In the title compound, C_19_H_16_F_3_N_3_, the dihedral angle between the naphthalene and quinoline ring systems is 14.58 (8)°. The hydrazone C—N—N=C—C chain is in an extended conformation and its mean plane is nearly coplanar with the quinoline plane [dihedral angle = 3.45 (9)°]. The bond angles within the phenyl ring show the almost additive influence of the two *para* substituents. In the crystal, weak π–π [centroid–centroid distances = 3.779 (2) and 3.718 (1) Å] and C—H⋯F directional inter­actions join the mol­ecules into centrosymmetric dimers, which are further connected into infinite zigzag chains propagating along *a*.

## Related literature

For second-order non-linear activity, see: Serbutoviez *et al.* (1995 ▶). For related structures, see: Jasinski *et al.* (2008 ▶); Yathirajan *et al.* (2007 ▶). For a description fo the Cambridge Structural Database, see: Allen (2002 ▶). For bond angles in mono-substituted phenyl rings, see: Domenicano (1988 ▶).
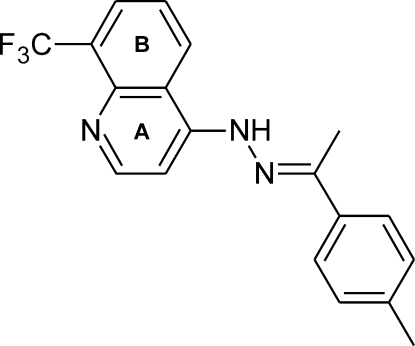


## Experimental

### 

#### Crystal data

C_19_H_16_F_3_N_3_
*M* *_r_* = 343.35
Monoclinic, 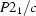

*a* = 8.2811 (9) Å
*b* = 14.8443 (15) Å
*c* = 13.5325 (15) Å
β = 90.601 (9)°
*V* = 1663.4 (3) Å^3^
*Z* = 4
Mo *K*α radiation
μ = 0.11 mm^−1^
*T* = 295 K
0.4 × 0.15 × 0.15 mm

#### Data collection

Oxford Diffraction Xcalibur Sapphire2 large Be window diffractometer
Absorption correction: multi-scan (*CrysAlis PRO*; Oxford Diffraction, 2009 ▶) *T*_min_ = 0.737, *T*_max_ = 1.000
8893 measured reflections
3391 independent reflections
2248 reflections with *I* > 2σ(*I*)
*R*_int_ = 0.021

#### Refinement

*R*[*F* ^2^ > 2σ(*F* ^2^)] = 0.049
*wR*(*F* ^2^) = 0.156
*S* = 1.12
3391 reflections
229 parameters
H-atom parameters constrained
Δρ_max_ = 0.25 e Å^−3^
Δρ_min_ = −0.19 e Å^−3^

### 

Data collection: *CrysAlis PRO* (Oxford Diffraction, 2009 ▶); cell refinement: *CrysAlis PRO*; data reduction: *CrysAlis PRO*; program(s) used to solve structure: *SIR92* (Altomare *et al.*, 1993 ▶); program(s) used to refine structure: *SHELXL97* (Sheldrick, 2008 ▶); molecular graphics: *Stereochemical Workstation Operation Manual* (Siemens, 1989 ▶) and *Mercury* (Macrae *et al.*, 2008 ▶); software used to prepare material for publication: *SHELXL97*.

## Supplementary Material

Crystal structure: contains datablocks I, global. DOI: 10.1107/S1600536810009475/dn2546sup1.cif

Structure factors: contains datablocks I. DOI: 10.1107/S1600536810009475/dn2546Isup2.hkl

Additional supplementary materials:  crystallographic information; 3D view; checkCIF report

## Figures and Tables

**Table 1:** Hydrogen-bond geometry (Å, °)

*D*—H⋯*A*	*D*—H	H⋯*A*	*D*⋯*A*	*D*—H⋯*A*
C7—H7⋯F91*A*^i^	0.93	2.50	3.336 (2)	150
C14—H14*C*⋯F91*C*^ii^	0.96	2.55	3.384 (2)	146
C17—H17⋯F91*C*^iii^	0.93	2.54	3.425 (3)	160

Symmetry codes: (i) 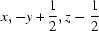; (ii) 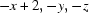; (iii) 
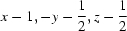
.

**Fig. 1.**
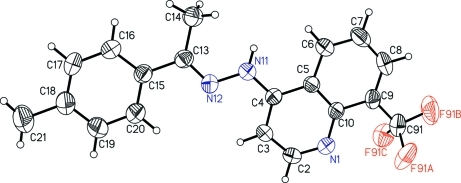
Anisotropic ellipsoid representation of the compound I together with atom labelling scheme. The ellipsoids are drawn at 50% probability level, hydrogen atoms are depicted as spheres with arbitrary radii.

**Fig. 2.**
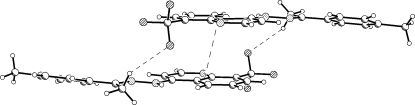
The centrosymmetric dimer of molecules I; π–π and C—H···F contacts are shown as dashed lines. are shown as dashed lines.

## supplementary crystallographic information

### Comment

Hydrazones constitute a class of compounds of general formula R_1_R_2_C═ N—NR_3_R_4_. Serbutoviez *et al.* (1995) have shown that some diaryl hydrazone derivatives show efficient second-order nonlinear activity. They connected the tendency to crystallize in Λ-shaped pairs with the possibility of the application for the frequency conversion but not for electrooptics. Here we present the structure of (*E*)-1-(4-methylphenyl)ethanone [8-(trifluoromethyl)quinolin-4-yl]hydrazone (I, Scheme 1); the crystal structures of two salts of similar (quinoline - phenyl) hydrazones have been reported recently: bis{4-[(2-hydroxybenzylidine)hydrazino]- 8-(trifluoromethyl)quinolinium} sulfate tetrahydrate (Yathirajan *et al.*, 2007) and bis{4-[(*Z*)—*N*'-(4-hydroxybenzylidene)- hydrazino]-8-(trifluoromethyl)- quinolinium} sulfate dihydrate (Jasinski *et al.*, 2008).

The overall conformation of the molecules of I can be described by the values of dihedral angles between the three planar fragments: the quinoline ring system (hereinafter A will denote pyridine ring, B - trifluoromethylphenyl ring, planar within 0.0076 (14) Å), the central extended C—N—N═ C—C chain (maximum deviation from the least-squares plane of 0.0158 (12) Å), and the phenyl ring (C, maximum deviation 0.021 (2) Å). While the first two fragments are almost coplanar, dihedral angle between the planes is only 3.45 (9)°, this fragment is significantly, by 14.5 (1)°, twisted with respect to the phenyl ring plane. Such conformation is rather typical; for 186 fragments found in 155 similar compunds (Ar_1_—N—N═C—Ar_2_) found in the Cambridge Structual Database [CSD, Conquest 5.31; Allen, 2002] the Ar_1_ plane is close to coplanarity with the central chain (mean value 5.9 (3)°, maximum 18.7°), while it is more twisted with respect to Ar_2_ plane (mean 17 (2)°, 33 examples of angles larger than 30°). The bond length pattern within the chain reflects the more single/double character of certain bonds. The bond angles within the phenyl ring are influenced by the presence of substituents; as expected for *p*-substitution, the influences are almost additive. The sum of values given by Domenicano (1988) or found in the CSD for mono-substituted phenyl rings are very close to the actual values in (I).

In the crystal the molecules are connected into dimers by π–π interactions: centroid-to-centroid distance between rings A and B (*2-x,-y,-z*) is 3.779 (2)Å with an offset of 22.1°, which gives the interplanar distance of 3.509Å (mean value). Distance between centroids of rings B and B(*2-x,-y,-z*) is 3.718 (1) Å, with interplanar distance of 3.516Å resulting in an offset of 19.0°. These dimers, in which there are additional C—H···F (Table 1) contacts (Fig. 2), are further connected into zig-zag chains (Λ-shaped) along *a* direction. It might be noted, that the N—H hydrogen atom is so hidden by the neighboring C6—H6 and C14 methyl hydrogen atoms that it can not be involved in any intermolecular interactions.

### Experimental

A solution of 4-hydrazino-8-(trifluoromethyl)quinoline (2.2 g, 10 mmole) and 4-methyl-acetophenone (10.2 mmole ) in 10 ml of ethanol was refluxed for 24 hrs under nitrogen atmosphere and in absence of light. The reaction mass was then cooled and the solid separated was collected by filtration and recrystallized from ethanol. M.P.: 449-451 K. Analysis found : C 66.41, H 4.67, N 12.20; C_19_H_16_F_3_N_3_ requires : C 66.48, H 4.70, N 12.24%.

### Refinement

Hydrogen atoms were located geometrically (*C*(methyl)-H 0.93 Å, C(ar)—H 0.96 Å, N—H 0.86 Å) and refined as a riding model; the U_iso_ values of H atoms were set at 1.2 (1.5 for methyl groups) times U_eq_ of their carrier atom.

### Crystal data

**Table:**

C_19_H_16_F_3_N_3_	*F*(000) = 712
*M**_r_* = 343.35	*D*_x_ = 1.371 Mg m^−^^3^
Monoclinic, *P*2_1_/*c*	Mo *K*α radiation, λ = 0.71073 Å
Hall symbol: -P 2ybc	Cell parameters from 4730 reflections
*a* = 8.2811 (9) Å	θ = 3.0–28.0°
*b* = 14.8443 (15) Å	µ = 0.11 mm^−^^1^
*c* = 13.5325 (15) Å	*T* = 295 K
β = 90.601 (9)°	Prism, yellow
*V* = 1663.4 (3) Å^3^	0.4 × 0.15 × 0.15 mm
*Z* = 4	

### Data collection

**Table:**

Oxford Diffraction Xcalibur Sapphire2 large Be window diffractometer	3391 independent reflections
Radiation source: Enhance (Mo) X-ray Source	2248 reflections with *I* > 2σ(*I*)
graphite	*R*_int_ = 0.021
Detector resolution: 8.1929 pixels mm^-1^	θ_max_ = 28.1°, θ_min_ = 3.0°
ω–scan	*h* = −10→10
Absorption correction: multi-scan (*CrysAlis PRO*; Oxford Diffraction, 2009)	*k* = −17→19
*T*_min_ = 0.737, *T*_max_ = 1.000	*l* = −17→13
8893 measured reflections	

### Refinement

**Table:**

Refinement on *F*^2^	Secondary atom site location: difference Fourier map
Least-squares matrix: full	Hydrogen site location: inferred from neighbouring sites
*R*[*F*^2^ > 2σ(*F*^2^)] = 0.049	H-atom parameters constrained
*wR*(*F*^2^) = 0.156	*w* = 1/[σ^2^(*F*_o_^2^) + (0.0897*P*)^2^] where *P* = (*F*_o_^2^ + 2*F*_c_^2^)/3
*S* = 1.12	(Δ/σ)_max_ = 0.001
3391 reflections	Δρ_max_ = 0.25 e Å^−^^3^
229 parameters	Δρ_min_ = −0.19 e Å^−^^3^
0 restraints	Extinction correction: *SHELXL97* (Sheldrick, 2008), Fc^*^=kFc[1+0.001xFc^2^λ^3^/sin(2θ)]^-1/4^
Primary atom site location: structure-invariant direct methods	Extinction coefficient: 0.019 (3)

### Special details

**Table:**

Geometry. All esds (except the esd in the dihedral angle between two l.s. planes) are estimated using the full covariance matrix. The cell esds are taken into account individually in the estimation of esds in distances, angles and torsion angles; correlations between esds in cell parameters are only used when they are defined by crystal symmetry. An approximate (isotropic) treatment of cell esds is used for estimating esds involving l.s. planes.
Refinement. Refinement of *F*^2^ against ALL reflections. The weighted *R*-factor wR and goodness of fit *S* are based on *F*^2^, conventional *R*-factors *R* are based on *F*, with *F* set to zero for negative *F*^2^. The threshold expression of *F*^2^ > σ(*F*^2^) is used only for calculating *R*-factors(gt) etc. and is not relevant to the choice of reflections for refinement. *R*-factors based on *F*^2^ are statistically about twice as large as those based on *F*, and *R*- factors based on ALL data will be even larger.

### Fractional atomic coordinates and isotropic or equivalent isotropic displacement parameters (Å^2^)

**Table:**

	*x*	*y*	*z*	*U*_iso_*/*U*_eq_	
N1	0.83992 (19)	0.01946 (10)	0.20074 (10)	0.0513 (4)	
C2	0.7602 (3)	−0.05642 (13)	0.20928 (14)	0.0597 (6)	
H2	0.7546	−0.0817	0.2721	0.072*	
C3	0.6830 (2)	−0.10262 (12)	0.13230 (13)	0.0537 (5)	
H3	0.6293	−0.1565	0.1444	0.064*	
C4	0.68734 (19)	−0.06746 (11)	0.03860 (12)	0.0401 (4)	
C5	0.77200 (18)	0.01539 (10)	0.02401 (11)	0.0370 (4)	
C6	0.7863 (2)	0.05794 (11)	−0.06808 (13)	0.0474 (5)	
H6	0.7370	0.0323	−0.1233	0.057*	
C7	0.8702 (2)	0.13543 (12)	−0.07816 (15)	0.0573 (5)	
H7	0.8792	0.1620	−0.1401	0.069*	
C8	0.9435 (2)	0.17588 (12)	0.00411 (16)	0.0553 (5)	
H8	1.0003	0.2295	−0.0034	0.066*	
C9	0.93256 (19)	0.13729 (11)	0.09527 (14)	0.0430 (4)	
C91	1.0141 (2)	0.18041 (12)	0.18190 (16)	0.0564 (5)	
F91A	0.91479 (15)	0.20118 (9)	0.25506 (10)	0.0827 (5)	
F91B	1.09032 (17)	0.25657 (8)	0.15776 (12)	0.0910 (5)	
F91C	1.12986 (14)	0.12807 (8)	0.22230 (9)	0.0708 (4)	
C10	0.84643 (19)	0.05542 (10)	0.10817 (12)	0.0388 (4)	
N11	0.61492 (17)	−0.10952 (9)	−0.04049 (11)	0.0469 (4)	
H11	0.6160	−0.0855	−0.0983	0.056*	
N12	0.54053 (17)	−0.19105 (9)	−0.02519 (10)	0.0451 (4)	
C13	0.47706 (18)	−0.23051 (12)	−0.10088 (13)	0.0409 (4)	
C14	0.4800 (2)	−0.19314 (13)	−0.20397 (13)	0.0536 (5)	
H14A	0.4451	−0.1315	−0.2031	0.080*	
H14B	0.4088	−0.2277	−0.2457	0.080*	
H14C	0.5879	−0.1964	−0.2290	0.080*	
C15	0.39938 (19)	−0.31859 (12)	−0.08073 (12)	0.0426 (4)	
C16	0.3532 (3)	−0.37659 (15)	−0.15437 (16)	0.0757 (7)	
H16	0.3689	−0.3603	−0.2199	0.091*	
C17	0.2841 (3)	−0.45844 (17)	−0.13344 (18)	0.0938 (9)	
H17	0.2546	−0.4961	−0.1855	0.113*	
C18	0.2571 (3)	−0.48652 (14)	−0.03884 (16)	0.0652 (6)	
C19	0.2963 (3)	−0.42716 (16)	0.03489 (17)	0.0790 (7)	
H19	0.2747	−0.4424	0.1001	0.095*	
C20	0.3673 (3)	−0.34517 (15)	0.01459 (15)	0.0711 (6)	
H20	0.3942	−0.3069	0.0666	0.085*	
C21	0.1827 (4)	−0.57785 (17)	−0.0168 (2)	0.0937 (8)	
H21A	0.1198	−0.5974	−0.0728	0.141*	
H21B	0.1145	−0.5730	0.0399	0.141*	
H21C	0.2669	−0.6208	−0.0036	0.141*	

### Atomic displacement parameters (Å^2^)

**Table:**

	*U*^11^	*U*^22^	*U*^33^	*U*^12^	*U*^13^	*U*^23^
N1	0.0716 (10)	0.0439 (9)	0.0383 (9)	−0.0069 (8)	0.0033 (7)	−0.0045 (7)
C2	0.0930 (14)	0.0510 (12)	0.0352 (11)	−0.0162 (11)	0.0069 (10)	0.0043 (8)
C3	0.0784 (13)	0.0422 (10)	0.0407 (11)	−0.0182 (9)	0.0096 (9)	0.0018 (8)
C4	0.0468 (9)	0.0359 (9)	0.0376 (10)	−0.0010 (7)	0.0063 (7)	−0.0037 (7)
C5	0.0413 (8)	0.0325 (9)	0.0374 (9)	0.0042 (7)	0.0063 (7)	0.0002 (7)
C6	0.0576 (11)	0.0425 (10)	0.0419 (11)	−0.0032 (8)	0.0007 (8)	0.0063 (8)
C7	0.0696 (12)	0.0489 (11)	0.0535 (12)	−0.0040 (10)	0.0015 (10)	0.0173 (9)
C8	0.0566 (11)	0.0326 (9)	0.0768 (15)	−0.0044 (8)	0.0033 (10)	0.0108 (9)
C9	0.0436 (9)	0.0305 (9)	0.0550 (11)	0.0029 (7)	0.0042 (8)	−0.0027 (8)
C91	0.0586 (11)	0.0383 (10)	0.0723 (14)	−0.0024 (9)	0.0007 (11)	−0.0079 (10)
F91A	0.0791 (8)	0.0770 (9)	0.0921 (10)	−0.0038 (6)	0.0074 (7)	−0.0479 (7)
F91B	0.1055 (10)	0.0536 (8)	0.1134 (11)	−0.0350 (7)	−0.0218 (8)	−0.0004 (7)
F91C	0.0662 (7)	0.0711 (8)	0.0748 (9)	0.0030 (6)	−0.0153 (6)	−0.0064 (6)
C10	0.0440 (9)	0.0318 (9)	0.0407 (10)	0.0044 (7)	0.0061 (7)	−0.0031 (7)
N11	0.0603 (9)	0.0418 (9)	0.0385 (8)	−0.0095 (7)	0.0008 (7)	0.0017 (6)
N12	0.0516 (8)	0.0389 (8)	0.0451 (9)	−0.0074 (6)	0.0035 (7)	−0.0030 (7)
C13	0.0398 (9)	0.0432 (10)	0.0398 (10)	0.0019 (7)	0.0027 (7)	−0.0035 (8)
C14	0.0549 (10)	0.0589 (12)	0.0468 (11)	−0.0128 (9)	−0.0026 (9)	0.0019 (9)
C15	0.0420 (9)	0.0446 (10)	0.0412 (10)	−0.0023 (7)	−0.0002 (7)	−0.0038 (8)
C16	0.1163 (18)	0.0696 (15)	0.0415 (12)	−0.0364 (14)	0.0040 (12)	−0.0073 (10)
C17	0.151 (2)	0.0727 (16)	0.0582 (15)	−0.0566 (16)	0.0012 (15)	−0.0164 (12)
C18	0.0776 (13)	0.0546 (13)	0.0633 (14)	−0.0191 (11)	−0.0049 (11)	0.0019 (10)
C19	0.1223 (19)	0.0664 (15)	0.0481 (13)	−0.0332 (14)	−0.0083 (13)	0.0116 (11)
C20	0.1085 (17)	0.0601 (13)	0.0446 (12)	−0.0282 (12)	−0.0088 (12)	−0.0011 (10)
C21	0.123 (2)	0.0671 (16)	0.0911 (19)	−0.0361 (15)	−0.0111 (16)	0.0132 (13)

### Geometric parameters (Å, °)

**Table:**

N1—C2	1.311 (2)	N11—H11	0.8600
N1—C10	1.363 (2)	N12—C13	1.287 (2)
C2—C3	1.397 (3)	C13—C15	1.484 (2)
C2—H2	0.9300	C13—C14	1.502 (2)
C3—C4	1.372 (2)	C14—H14A	0.9600
C3—H3	0.9300	C14—H14B	0.9600
C4—N11	1.372 (2)	C14—H14C	0.9600
C4—C5	1.430 (2)	C15—C16	1.369 (3)
C5—C6	1.403 (2)	C15—C20	1.377 (2)
C5—C10	1.420 (2)	C16—C17	1.374 (3)
C6—C7	1.351 (2)	C16—H16	0.9300
C6—H6	0.9300	C17—C18	1.367 (3)
C7—C8	1.398 (3)	C17—H17	0.9300
C7—H7	0.9300	C18—C19	1.368 (3)
C8—C9	1.364 (3)	C18—C21	1.520 (3)
C8—H8	0.9300	C19—C20	1.380 (3)
C9—C10	1.421 (2)	C19—H19	0.9300
C9—C91	1.491 (3)	C20—H20	0.9300
C91—F91A	1.330 (2)	C21—H21A	0.9600
C91—F91B	1.337 (2)	C21—H21B	0.9600
C91—F91C	1.345 (2)	C21—H21C	0.9600
N11—N12	1.3746 (18)		
			
C2—N1—C10	116.24 (15)	N12—N11—H11	120.8
N1—C2—C3	125.66 (17)	C13—N12—N11	117.43 (14)
N1—C2—H2	117.2	N12—C13—C15	115.40 (15)
C3—C2—H2	117.2	N12—C13—C14	124.10 (16)
C4—C3—C2	119.09 (16)	C15—C13—C14	120.50 (15)
C4—C3—H3	120.5	C13—C14—H14A	109.5
C2—C3—H3	120.5	C13—C14—H14B	109.5
N11—C4—C3	122.16 (15)	H14A—C14—H14B	109.5
N11—C4—C5	119.64 (15)	C13—C14—H14C	109.5
C3—C4—C5	118.19 (15)	H14A—C14—H14C	109.5
C6—C5—C10	118.96 (15)	H14B—C14—H14C	109.5
C6—C5—C4	123.75 (15)	C16—C15—C20	116.53 (17)
C10—C5—C4	117.29 (14)	C16—C15—C13	122.61 (17)
C7—C6—C5	121.40 (17)	C20—C15—C13	120.85 (16)
C7—C6—H6	119.3	C15—C16—C17	121.3 (2)
C5—C6—H6	119.3	C15—C16—H16	119.3
C6—C7—C8	120.31 (17)	C17—C16—H16	119.3
C6—C7—H7	119.8	C18—C17—C16	122.4 (2)
C8—C7—H7	119.8	C18—C17—H17	118.8
C9—C8—C7	120.48 (16)	C16—C17—H17	118.8
C9—C8—H8	119.8	C17—C18—C19	116.54 (19)
C7—C8—H8	119.8	C17—C18—C21	121.8 (2)
C8—C9—C10	120.57 (17)	C19—C18—C21	121.7 (2)
C8—C9—C91	119.79 (16)	C18—C19—C20	121.4 (2)
C10—C9—C91	119.63 (16)	C18—C19—H19	119.3
F91A—C91—F91B	106.44 (16)	C20—C19—H19	119.3
F91A—C91—F91C	105.95 (17)	C15—C20—C19	121.71 (19)
F91B—C91—F91C	104.57 (15)	C15—C20—H20	119.1
F91A—C91—C9	113.97 (16)	C19—C20—H20	119.1
F91B—C91—C9	112.45 (17)	C18—C21—H21A	109.5
F91C—C91—C9	112.74 (15)	C18—C21—H21B	109.5
N1—C10—C5	123.52 (14)	H21A—C21—H21B	109.5
N1—C10—C9	118.20 (15)	C18—C21—H21C	109.5
C5—C10—C9	118.28 (15)	H21A—C21—H21C	109.5
C4—N11—N12	118.44 (14)	H21B—C21—H21C	109.5
C4—N11—H11	120.8		
			
C10—N1—C2—C3	0.1 (3)	C4—C5—C10—C9	179.39 (13)
N1—C2—C3—C4	−0.4 (3)	C8—C9—C10—N1	179.64 (15)
C2—C3—C4—N11	179.62 (17)	C91—C9—C10—N1	0.8 (2)
C2—C3—C4—C5	0.2 (3)	C8—C9—C10—C5	−0.3 (2)
N11—C4—C5—C6	0.1 (2)	C91—C9—C10—C5	−179.13 (15)
C3—C4—C5—C6	179.55 (17)	C3—C4—N11—N12	−2.2 (2)
N11—C4—C5—C10	−179.21 (14)	C5—C4—N11—N12	177.15 (13)
C3—C4—C5—C10	0.2 (2)	C4—N11—N12—C13	−178.20 (15)
C10—C5—C6—C7	0.6 (3)	N11—N12—C13—C15	179.54 (13)
C4—C5—C6—C7	−178.79 (16)	N11—N12—C13—C14	0.2 (2)
C5—C6—C7—C8	−0.8 (3)	N12—C13—C15—C16	−167.60 (19)
C6—C7—C8—C9	0.6 (3)	C14—C13—C15—C16	11.8 (3)
C7—C8—C9—C10	0.0 (3)	N12—C13—C15—C20	13.7 (2)
C7—C8—C9—C91	178.86 (17)	C14—C13—C15—C20	−166.96 (18)
C8—C9—C91—F91A	122.12 (19)	C20—C15—C16—C17	−2.4 (4)
C10—C9—C91—F91A	−59.0 (2)	C13—C15—C16—C17	178.8 (2)
C8—C9—C91—F91B	0.9 (2)	C15—C16—C17—C18	0.2 (4)
C10—C9—C91—F91B	179.75 (15)	C16—C17—C18—C19	2.7 (4)
C8—C9—C91—F91C	−117.05 (18)	C16—C17—C18—C21	−179.1 (3)
C10—C9—C91—F91C	61.8 (2)	C17—C18—C19—C20	−3.3 (4)
C2—N1—C10—C5	0.4 (3)	C21—C18—C19—C20	178.5 (2)
C2—N1—C10—C9	−179.55 (16)	C16—C15—C20—C19	1.7 (3)
C6—C5—C10—N1	−179.90 (15)	C13—C15—C20—C19	−179.5 (2)
C4—C5—C10—N1	−0.5 (2)	C18—C19—C20—C15	1.2 (4)
C6—C5—C10—C9	0.0 (2)		

### Hydrogen-bond geometry (Å, °)

**Table:**

*D*—H···*A*	*D*—H	H···*A*	*D*···*A*	*D*—H···*A*
C7—H7···F91A^i^	0.93	2.50	3.336 (2)	150
C14—H14C···F91C^ii^	0.96	2.55	3.384 (2)	146
C17—H17···F91C^iii^	0.93	2.54	3.425 (3)	160

Symmetry codes: (i) *x*, −*y*+1/2, *z*−1/2; (ii) −*x*+2, −*y*, −*z*; (iii) *x*−1, −*y*−1/2, *z*−1/2.
